# Determination of Carbohydrate Structure Recognized by Prostate-specific F77 Monoclonal Antibody through Expression Analysis of Glycosyltransferase Genes*

**DOI:** 10.1074/jbc.M114.559047

**Published:** 2014-04-21

**Authors:** Motohiro Nonaka, Michiko N. Fukuda, Chao Gao, Zhen Li, Hongtao Zhang, Mark I. Greene, Donna M. Peehl, Ten Feizi, Minoru Fukuda

**Affiliations:** From the ‡ Tumor Microenvironment Program, Cancer Center, Sanford-Burnham Medical Research Institute, La Jolla, California 92037,; the ¶ Department of Pathology and Laboratory Medicine, University of Pennsylvania, Philadelphia, Pennsylvania 19104-6082,; the ‖ Department of Urology, Stanford University School of Medicine, Stanford, California 94305-5118, and; the § Glycosciences Laboratory, Department of Medicine, Imperial College London, Hammersmith Campus, Du Cane Road, London W12 0NN, United Kingdom

**Keywords:** Antigen, Carbohydrate Biosynthesis, Carbohydrate Structure, Glycobiology, Glycosyltransferase, Biomarker of Epithelial Cancer Cell, Cancer-associated Carbohydrate Antigen

## Abstract

This study reports the determination of the carbohydrate epitope of monoclonal antibody F77 previously raised against human prostate cancer PC-3 cells (Zhang, G., Zhang, H., Wang, Q., Lal, P., Carroll, A. M., de la Llera-Moya, M., Xu, X., and Greene, M. I. (2010) *Proc. Natl. Acad. Sci. U. S. A.* 107, 732–737). We performed a series of co-transfections using mammalian expression vectors encoding specific glycosyltransferases. We thereby identified branching enzymes and FUT1 (required for Fucα1→2Gal linkage) as being essential for F77 antigen formation. When immortalized normal prostate 267B1 cells were transfected with FUT1 alone, cells showed weak expression of F77 antigen. By contrast, cells co-transfected with FUT1 plus either GCNT1, GCNT2, or GCNT3 (an enzyme required to form GlcNAcβ1→6Gal/GalNAc) showed robust F77 antigen expression, suggesting that F77 specifically binds to Fucα1→2Galβ1→4GlcNAcβ1→6Gal/GalNAc. RT-PCR for FUT1, GCNT1, GCNT2, and GCNT3 showed that F77-positive cell lines indeed express transcripts encoding FUT1 plus one GCNT. F77-positive prostate cancer cells transfected with siRNAs targeting FUT1, GCNT2, and GCNT3 showed significantly reduced F77 antigen, confirming the requirement of these enzymes for epitope synthesis. We also found that hypoxia induces F77 epitope expression in immortalized prostate RWPE1 cells, which express F77 antigen moderately under normoxia but at an elevated level under hypoxia. Quantitative RT-PCR demonstrated up-regulation of FUT1, GCNT2, and GCNT3 transcripts in RWPE1 cells under hypoxia, suggesting that hypoxia up-regulates glycosyltransferase expression required for F77 antigen synthesis. These results define the F77 epitope and provide a potential mechanism for F77 antigen synthesis in malignant prostate cancer.

## Teaser

**Background:** Monoclonal antibody F77 raised against the PC-3 cells recognizes human prostate cancers.

**Results:** Co-transfections with glycosyltransferase genes showed that FUT1 and one of GCNT1, GCNT2, or GCNT3 are necessary for F77 antigen expression in mammalian cells.

**Conclusion:** F77 antigen is expressed on glycan structures composed of Fucα1→2Galβ1→4GlcNAcβ1→6Gal/GalNAc.

**Significance:** Defining the F77 epitope provides a basis for clinical applications of this antibody.

## Introduction

Epithelial cells are covered by a thick layer of glycans called the glycocalyx, whose carbohydrate structures undergo structural changes following transformation to adenocarcinoma (1–11). When tissues or cells from cancer patients are used as immunogens, immunized mice often generate monoclonal antibodies (mAbs) specific to cancer cell surface carbohydrate antigens. Immunoassay of oligosaccharides and glycoproteins is difficult as a large amount of purified material is required as an inhibitor in binding assays. The specificities of monoclonal anti-carbohydrate antibodies have been largely determined using glycolipids (12) or neoglycolipids (13) as lipid-linked glycans bound to plastic plates or displayed on thin layer chromatography can readily be assayed for binding by antibodies. Highly specific mAbs are useful for both prognosis of cancer patients (14, 15) and molecular analysis of embryonic development and cellular differentiation (16).

Among numerous anti-carbohydrate mAbs raised against malignant tumor tissues, several reportedly kill targeted cells through antibody-dependent cellular cytotoxicity and antibody-dependent phagocytosis by macrophages (17–20). The mAb F77, which was produced by immunizing mice with the prostate cancer cell line PC-3 (20), is one such cytotoxic antibody. F77 preferentially binds to prostate cancer *versus* normal prostate tissue in a manner correlating with tumor grade. Remarkably, when F77 was injected intraperitoneally into mice bearing human prostate tumor xenografts, it effectively suppressed tumor outgrowth *in vivo* (20).

A previous study indicated that mAb F77 binds to glycolipids prepared from PC-3 cells, but the epitope recognized by this antibody remained undetermined (20). Because of its clinical potential, we have further investigated the epitope specifically recognized by the mAb F77. In the accompanying article (43), glycan array and mass spectrometric approaches have been used to characterize the F77 antigen. In this article, we have undertaken a genetic approach similar to that which we employed to determine novel carbohydrate structures recognized by other mAbs (19, 21), and we have performed transfections of an array of glycosyltransferse genes to identify the key enzymes involved in biosynthesis of the F77 antigen. Our data also demonstrated that glycosyltransferase genes functioning in F77 antigen synthesis are enhanced by hypoxia, linking F77 antigen expression to prostate tumor malignancy.

## EXPERIMENTAL PROCEDURES

### 

#### 

##### Cell Culture

PC-3 cells were cultured in Ham's F-12 medium (Mediatech, Inc.). LNCaP cells were cultured in RPMI 1640 medium (Mediatech). DU 145 cells were cultured in Eagle's minimum essential medium (Thermo Scientific). 267B1, HEK293, and CHO cells were cultured in Dulbecco's modified Eagle's high glucose medium (Thermo Scientific). All media were supplemented with 10% fetal calf serum. RWPE-1 and RWPE-2 cells were cultured in keratinocyte serum-free medium (Invitrogen). Cells were also cultured in the presence of 20 μg/ml dl-PPMP^3^ (Santa Cruz Biotechnology) for 48 h to inhibit glycosphingolipid synthesis or 5 mm benzyl 2-acetamido-2-deoxy-α-d-galactopyranoside (Bz-GalNAc) (Sigma) for 48 h to inhibit *O-*glycan synthesis. Synthetic oligosaccharides were provided by the Consortium for Functional Glycomics and were designated as TrlI7 (H tri type 2 referred to in Fig. 4, legend), Te222 (A tetra type 2), Te223 (B tetra type 2), Tr59 (Le^X^). For hypoxia experiments, tissue culture plates were placed in a hypoxia incubator chamber (Stemcell Technologies) containing 95% nitrogen, 5% carbon dioxide and incubated at 37 °C for 48 h.

##### Antibodies and Plasmid Vectors

F77 mAb was purified from culture supernatant of F77–129 hybridoma cells as described previously (22). Anti-Le^Y^ antibody (clone AH6) was provided by Dr. S. Hakomori (23). Mammalian expression vectors for CHST1 (keratan sulfate Gal6-sulfotransferase), CH6ST2 (GlcNAc-6-sulfotransferase-1), CHST4 (L-selectin ligand sulfotransferase), Chst5 (mouse intestinal GlcNAc-6-sulfotransferase), CHST6 (human corneal keratan sulfate GlcNAc-6-sulfotransferase), and GAL3ST3 (galactose-3-*O*-sulfotransferase 3) were previously reported (21). GAL3ST1 (galactose-3-*O*-sulfotransferase 1) was purchased from Origene. Glycosyltransferase genes encoding FUT1 (α1,2-fucosyltransferase), FUT3 (α3/4-fucosyltransferase), B3GNT3 (core 1 extension enzyme), B3GNT6 (core 3 synthase), GCNT1 (core 2 *N*-acetylglucosaminyltransferase, lymphocyte type), GCNT3 (core 2 *N*-acetylglucosaminyltransferase, mucin-type), B3GNT2 (polylactosamine extension enzyme), B3GNT7 (sulfated polylactosamine extension enzyme), and B3GALT5 (type 1 galactosyltransferase) were described previously (21). cDNA encoding GCNT2 (polylactosamine branching *N*-acetylglucosaminyltransferase) was cloned in our laboratory, and the expression vector was prepared as described previously (24). Expression vectors for blood group A and B were kindly provided by Dr. F. Yamamoto, Institute of Predictive and Personalized Medicine of Cancer (Barcelona, Spain).

##### Transfection of Plasmid Vectors and siRNA Oligonucleotides

267B1, HEK293, and CHO cells were cultured on glass coverslips in 24-well plates. Transfection of plasmid DNA was performed using Lipofectamine 2000 (Invitrogen). A total of 0.2 μg of expression vector DNAs containing equal amounts of plasmid by weight was used for co-transfection. Cells were incubated with transfection medium for 4 h in the presence of 10% fetal calf serum, followed by incubation with fresh culture medium. For siRNA transfections, respective Silencer Select siRNAs targeting FUT1, GCNT2, or GCNT3 were purchased from Invitrogen. A total of 5 pmol of siRNA was mixed with Lipofectamine 2000 reagent and added to each well with cultured host cells.

##### Immunocytochemistry and Fluorescence Staining

At 18–48 h post-transfection, coverslips were collected, and cells were fixed with 4% paraformaldehyde in PBS at room temperature for 15 min. Cells were then washed with PBS and incubated with 0.3% hydrogen peroxide in methanol at room temperature for 30 min. Immunohistochemistry was performed using Vectastain Elite ABC and 3,3′-diaminobenzidine staining kits (Vector Laboratories) according to the manufacturer's instructions. F77 antibody was used at 5 μg/ml. Hematoxylin served as counterstain. For the ImageJ quantification, three images of a 635 × 635-μm square were randomly chosen in each transfectant. The images were subjected to a color threshold to cover only the brown area corresponding to the F77 epitope. Integrated density, which represents the sum of the pixel values inside the area, was then calculated by the software, and the control sample was set as 1 for relative intensity. S.D. value was determined by the data from the three images. For fluorescence staining, fixed cells were stained with biotinylated *Ulex europaeus* agglutinin-I (UEA) lectin (Vector Laboratories), followed by Texas Red-conjugated streptavidin (Pierce). After blocking by the avidin/biotin blocking kit (Vector Laboratories), cells were stained by F77, followed by biotinylated anti-mouse IgG and fluorescein-conjugated streptavidin (Vector Laboratories).

##### Flow Cytometry

Cells were trypsinized, washed twice with PBS, and then fixed with 4% paraformaldehyde in PBS at room temperature for 15 min. Following washing with PBS, cells were blocked with 10% goat serum for 30 min. Incubation with mAb F77 (5 μg/ml) or anti-Le^Y^ antibody (1:800 dilution) was performed at room temperature for 30 min. After washing cells with PBS, cells were incubated with Alexa Fluor 488-labeled anti-mouse IgG antibody for F77 and anti-mouse IgM for AH6. Fluorescence-labeled cells were analyzed using FACSCalibur. For mean fluorescence intensity, each value of the geometric mean was calculated by CellQuest software, and the geometric mean of mock siRNA was set as 100.

##### ELISA Inhibition Assay Using Synthetic Oligosaccharides

PC-3 cells were grown in 96-well culture plates, fixed with 4% paraformaldehyde in PBS, and treated with methanol containing 0.3% hydrogen peroxide overnight at 4 °C. After blocking with Superblock Blocking buffer (Thermo Scientific)/PBS (1:1), diluted (0.1 μg/ml) F77 antibody plus serially diluted oligosaccharides (1.56–10 mm) received from the Consortium for Functional Glycomics were added. Their sequences are given in the legend to Fig. 4. After incubation at room temperature for 1 h, wells were washed with PBST and reacted with diluted (1:2000) peroxidase-conjugated anti-mouse IgG antibody for 1 h. Wells were then washed with PBST and reacted with 3,3′-tetramethylbenzidine substrate solution (eBioscience). The peroxidase reaction was stopped by adding 2.5 n sulfuric acid to 0.5 n at a final concentration, and the absorbance at 450 nm was recorded using a plate reader (Molecular Devices).

##### RT-PCR and Quantitative PCR

Total RNA was extracted from cells using TRIzol reagent (Invitrogen), and cDNA was synthesized using Superscript III reverse transcriptase (Invitrogen) according to the manufacturer's instruction. For RT-PCR, the following primer sets were used: FUT1, CCGGTTTGGTAATCAGATGG and CTCAAGTCCGCGTACTCCTC; GCNT1, TGCTTCCTCCACTCGAAACA and TGTCTTGTGCCCACTCCATC; GCNT2, ATCCTGGACTGGAAACCTCAG and CTTGTTAGCAAACAGGCTTGG; and GCNT3, GGAGTCCAGGGAATCTCAAAG and GAGGGAGAGGTAGTGGGTGTC. RT-PCR was performed for 35 cycles in the transcripts, β-actin, FUT1, GCNT2, and GCNT3. Following RNA isolation and cDNA synthesis, quantitative PCR with SYBR Green was performed for each transcript. The reaction was run and analyzed using a 7900HT Fast Real Time PCR system (Applied Biosystems). The following primer sets were used for PCR: FUT1, GCAGCTTCACGACTGGATGT and CTCTCTGCGGATCTGTTCCC; GCNT1, AGTAGGGAGTATGTGGGGTATGT and CTGGCAACTGCTTGCATGT; GCNT2, TGTTCCTGGCTCTATGCCAAA and TTAGCAAACAGGCTTGGTGAAT; GCNT3, TCAAAGAGGCGGTCAAAGCAA and GCATAAACCACCCGAACCAG; FUT7, TTCGTGCATGTGGATGACTTTGGC and AGCGTTGGTATCGGCTCTCATTCA.

## RESULTS

### 

#### 

##### Identification of Glycosyltransferases Required for F77 Antigen Biosynthesis

**FIGURE 1.**
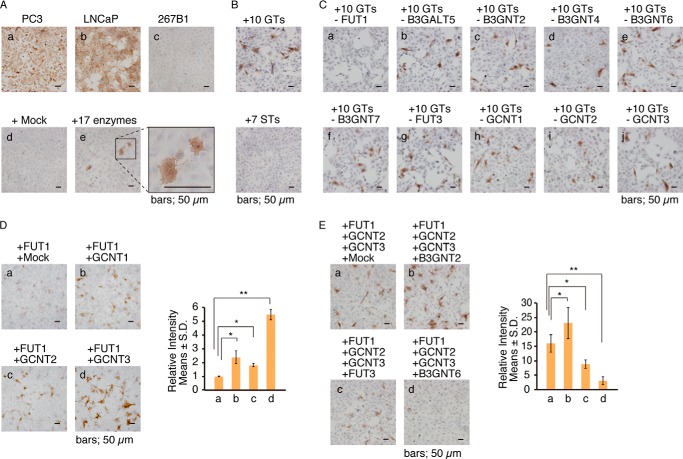
**Immunocytochemistry of 267B1 cells transfected with various glycosyltransferase constructs.** *A*, immunocytochemistry of PC-3 (*panel a*), LNCaP (*panel b*), and 267B1 (*panels c--e*) cells for F77. Immunostaining is shown without transfection (*panel c*), after mock transfection (*panel d*), and after transfection with a mixture of 10 GTs and 7 STs (*panel e*). Antigen-positive cells were detected by peroxidase-conjugated anti-mouse IgG antibody followed by peroxidase color reaction using the peroxidase substrate 3,3′-diaminobenzidine. Counterstaining was with hematoxylin. *B–E*, immunocytochemistry of 267B1 cells for F77. *B*, cells were transfected either with 10 GTs or with 7 STs. *C*, cells were transfected with a mixture of 9 GTs remaining after removal of the indicated enzyme from the original group of 10. *D* and *E*, cells were transfected with indicated GT(s). In each experiment, the total amount of plasmid DNA was adjusted to 0.2 μg. Positive immunostaining signals were quantitated using the ImageJ program (National Institutes of Health). *Error bars* indicate S.D. Unpaired two-tailed Student's *t* test was used for statistical analysis. Each transfection experiment was performed at least twice. A *p* value of less than 0.05 was considered significant (*). **, *p* < 0.01.

As reported previously (20), prostate cancer cell lines PC-3 and LNCaP express F77 antigen and are stained strongly by F77 mAb (Fig. 1*A*, *panels a* and *b*). By contrast, immortalized neonatal human prostate epithelial 267B1 cells are not stained by this antibody (Fig. 1*A*, *panel c*). To identify enzyme(s) required for biosynthesis of F77 antigen, 267B1 cells were transiently transfected with a mixture of 17 plasmid vectors encoding 10 glycosyltransferases (GTs) and 7 sulfotransferases (STs), in a manner similar to that described previously (21). We identified a small number of cells robustly stained by F77, although no positives were identified in mock transfectants (Fig. 1*A*, *panels e* and *d*). When 267B1 cells were transfected using either a mixture of all 10 GTs or all 7 STs, positive cells were detected in the GT-transfected but not ST-transfected cells (Fig. 1*B*), indicating that F77 antigen synthesis requires GTs and not STs.

To identify the enzyme most critical for F77 antigen biosynthesis among the 10 GTs, 267B1 cells were transfected with respective mixtures of 9 GTs from which one of the original 10 had been removed (Fig. 1*C*). This analysis showed that when a mixture lacked FUT1 (required for biosynthesis of Fucα1→2Gal linkage), there was a lack of F77 antigen-positive cells (Fig. 1*C*, *panel a*). F77 antigen positivity persisted following subtraction of each of the other enzymes from the mixture of 10 (Fig. 1*C*, *panels b–j*), indicating that FUT1 is necessary for F77 antigen biosynthesis. As a test of sufficiency, 267B1 cells were transfected by FUT1 alone and found to exhibit weak F77 positivity (Fig. 1*D*, *panel a*), suggesting that, although FUT1 is essential, additional enzyme(s) are required for robust F77 antigen expression. To identify those enzyme(s), we performed transfection with FUT1 plus candidate constructs and identified GCNT1, GCNT2, and GCNT3 (Fig. 1*D*, *panels b–d*) as enzymes enhancing the synthesis of F77 antigen.

Further analysis showed that 267B1 cells transfected with a mixture of FUT1 and both GCNT2 and -3 exhibited strong F77 antigen positivity (Fig. 1*E*, *panel a*). GCNT1 was excluded from this experiment because GCNT3 has broader enzymatic specificity (formation of core2/core4) than GCNT1 (core2 formation only) and overlaps the function of GCNT1. In addition, inclusion of B3GNT2 in the GT mixture significantly increased F77 staining intensity (Fig. 1*E*, *panel b*). B3GNT2 elongates poly-*N*-acetyllactosamine chains, suggesting that linear elongation of poly-*N*-acetyllactosamine provides additional acceptors for GCNT2, increasing the number of F77 epitope structures. We also found that addition of FUT3 to a mixture of FUT1, GCNT2, and GCNT3 significantly reduced levels of F77 antigen (Fig. 1*E*, *panel c*), suggesting that FUT3, which adds the Fucα1→3/4 linkage on GlcNAc, forms Lewis X antigen or converts H antigen to Le^Y^ antigen. Thus, we conclude that F77 does not recognize Le^X^ or Le^Y^ antigens. Furthermore, addition of B3GNT6 or core-3 *O-*glycan synthase significantly inhibited F77 antigen synthesis (Fig. 1*E*, *panel d*), suggesting that formation of core 3 structures reduces the number of core 2 structures generated by GCNT3.

**FIGURE 2.**
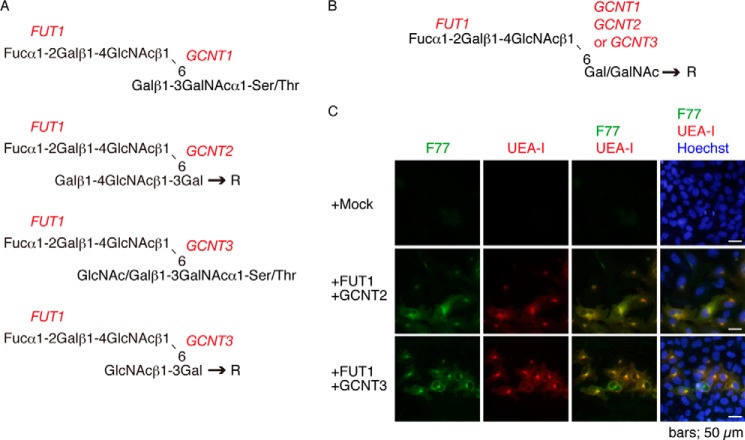
*A,* proposed synthetic pathways by FUT1 and GCNT1/2/3. Transfection analysis shows that enzymes essential for mammalian cells to synthesize F77 antigen are FUT1 plus either GCNT1, GCNT2, or GCNT3. *B,* proposed sequence of F77 epitope. Transfection analysis suggests that F77 recognizes a Fucα1–2Galβ1–4GlcNAc terminal structure linked to Gal or GalNAc through a GlcNAcβ1–6 linkage. *C,* fluorescence staining of 267B1 transfectant cells by F77 and UEA-I lectin. 267B1 cells were transfected with a mixture of indicated GTs and stained for F77. Counterstaining was with Hoechst.

GCNT2 catalyzes branching of poly-*N*-acetyllactosamine type glycans or blood group I antigen (24), and GCNT3 mainly synthesizes the core 2 or core 4 branch of *O-*glycans (GlcNAc/Galβ1–3(GlcNAcβ1–6)GalNAc) in mucin glycoproteins (25) (Fig. 2*A*). Collectively, these observations suggest that F77 antigen is determined by the Fucα1–2Gal sequence presented by GlcNAcβ1→6 branching on Gal/GalNAc residues, or Galβ1→4GlcNAcβ1→3(Fucα1→2Galβ1→4GalNAcβ1→6)Gal/GalNAc→R, as shown in Fig. 2*B*. Indeed, UEA-I lectin, which recognizes the α1,2-linked fucose residue, stained F77 antigen-expressing cells (Fig. 2*C*). Overall, these results also strongly suggest that either FUT1 and GCNT2 or FUT1 and GCNT3 are required for biosynthesis of F77 antigen in 267B1 cells.

##### Reactivity of F77 mAb with Blood Group ABO Antigens

**FIGURE 3.**
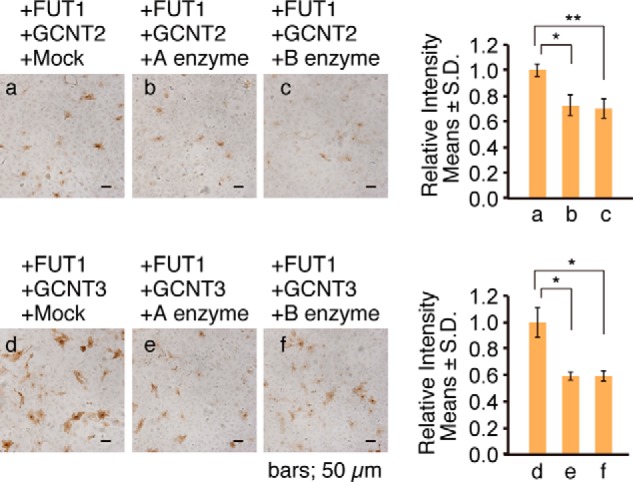
**Immunocytochemistry of 267B1 cells transfected with plasmid vectors encoding respective blood group A and B enzymes.** 267B1 cells were transfected with a mixture of indicated GTs and stained for F77. *Error bars* indicate S.D. A two-tailed unpaired Student's *t* test was used for statistical analysis. A *p* value less than 0.05 was considered significant (*). **, *p* < 0.01.

As the proposed F77 epitope contains blood group H or O antigen, we asked whether F77 mAb binds to cells expressing the blood group A and/or B antigens. A enzyme and B enzyme add GalNAcα1→3 and Galα1→3 linkages, respectively, to terminal Gal residues of H antigen (26, 27). When 267B1 cells were transfected with A enzyme together with either FUT1 plus GCNT2 or FUT1 plus GCNT3 constructs, the relative number of F77-positive cells significantly decreased (Fig. 3, *a–c*). Equivalent results were seen following similar assays using B enzyme (Fig. 3, *d–f*). These results suggest that F77 mAb binds to the blood group H antigen and binds less strongly to blood group A or B antigens.

**FIGURE 4.**
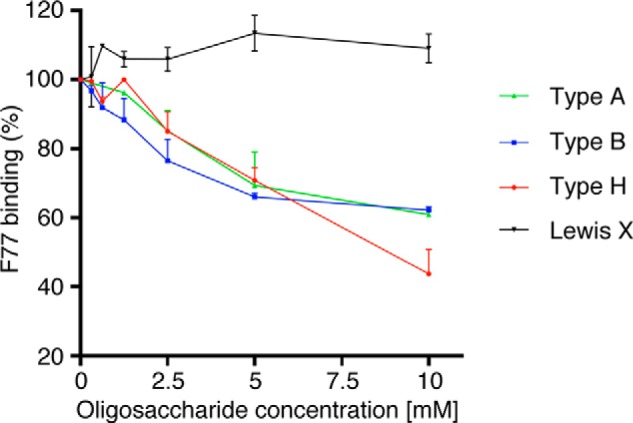
**ELISA inhibition assay using chemically synthesized blood group ABO oligosaccharides.** PC-3 cells were grown in 96-well plates and reacted with F77 in the presence of oligosaccharides at concentrations ranging from 0 to 10 mm. Antibody binding was assessed using an HRP-conjugated second antibody followed by incubation with peroxidase substrate. Oligosaccharides used in this experiment were type 2H trisaccharide, Fucα1→2Galβ1→4GlcNAc-CH_2_CH_2_N_3_; type 2A tetrasaccharide, Fucα1→2(GalNAcα1→3)Galβ1→4GlcNAc-CH_2_CH_2_N_3_; type 2B tetrasaccharide, Fucα1→2(Galα1→3)Galβ1→4GlcNAc-CH_2_CH_2_N_3_; and Lewis X trisaccharide, Galβ1→4(Fucα1→3)GlcNAc-CH_2_CH_2_N_3_.

To further compare the F77 reactivity with blood group ABH antigens, we performed ELISA inhibition assays using chemically synthesized oligosaccharides. When fixed PC-3 cells were incubated with F77 antibody in the presence of type H trisaccharide or type A or B tetrasaccharide, antibody binding was inhibited at high concentrations of the oligosaccharides (Fig. 4). The inhibitory effect of type H trisaccharide at 10 mm was greater (>50% inhibition) than that of type A and B tetrasaccharide (<40% inhibition), suggesting that F77 antibody prefers a type H structure over A or B structures.

##### Expression Analysis of Glycosyltransferases Functioning in F77 Antigen Biosynthesis

**FIGURE 5.**
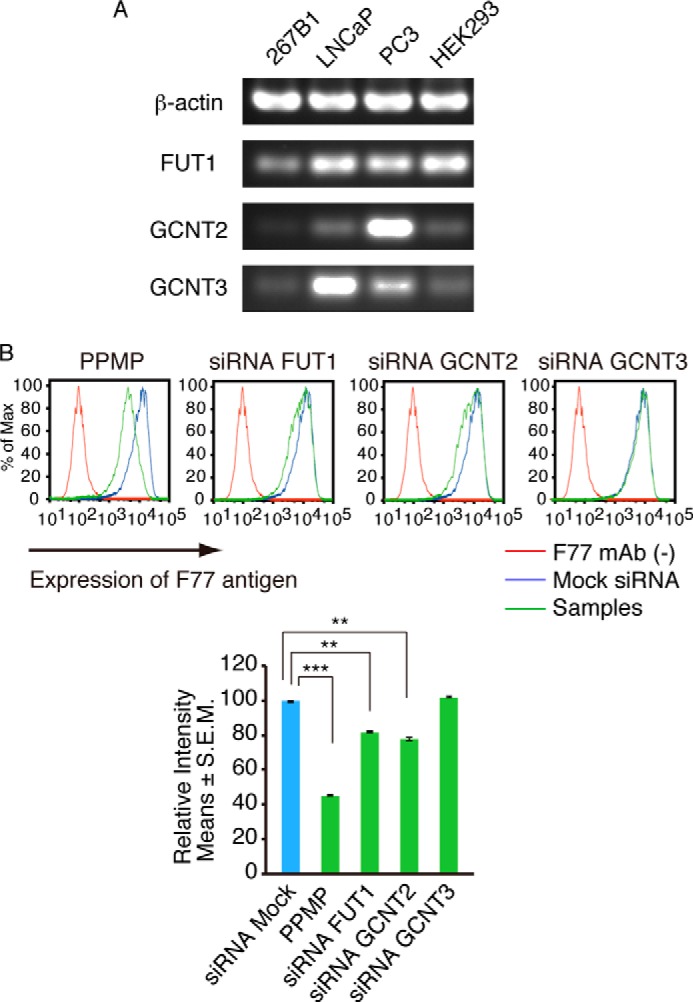
**RT-PCR and knockdown of genes catalyzing F77 antigen synthesis in various cell lines.** *A*, agarose gel showing RT-PCR of transcripts encoding respective enzymes functioning in F77 epitope synthesis. RNA isolated from indicated lines was subjected to RT-PCR using primers specific to each transcript. *B,* effect of gene knockdown on F77 epitope synthesis in PC-3 cells. Cells were transfected with siRNAs for FUT1, GCNT2, or GCNT3. *Histogram* of mock siRNA (*blue lines*) was overlaid with siRNA-transfected samples (*green lines*). Data without F77 are shown as *red lines*. PPMP, an inhibitor of glycosphingolipid synthesis, served as a positive control. Quantitative analyses of flow cytometry are shown in *bar graphs* on the *right. Error bars* indicate S.E. Population comparison was performed using Flowjo software. The data shown are representative of two experiments with similar results. A *p* value less than 0.05 was considered significant (*). **, *p* < 0.01; ***, *p* < 0.001.

We next used RT-PCR to compare expression of mRNAs encoding FUT1, GCNT1, GCNT2, and GCNT3 in F77-positive and -negative cell lines (Fig. 5*A*). F77 antigen-negative 267B1 cells did not express GCNT2 or GCNT3 and only very low levels of FUT1. HEK293 cells, which are also F77-negative (20), expressed FUT1 but not GCNT2 or GCNT3. By contrast, F77-positive PC-3 cells expressed high levels of FUT1 and GCNT2, and F77-positive LNCaP cells expressed FUT1 plus GCNT3. These results show that F77 epitope-positive cell lines indeed express transcripts for FUT1 plus one GCNT, confirming our identification of enzymes required for epitope synthesis in F77-positive cell lines.

##### FUT1 and GCNT2 Are Sufficient for Synthesis of F77 Antigen in PC-3 Cells

We next asked whether FUT1 and GCNT2 are sufficient for F77 epitope synthesis in PC-3 cells using gene knockdown. Flow cytometry analysis of PC-3 cells transfected with FUT1 siRNA showed significantly reduced levels of F77 antigen (Fig. 5*B*). GCNT2 knockdown PC-3 cells also showed reduced F77 antigen levels, whereas F77 antigen was not altered following transfection with mock siRNA or with GCNT3 siRNA. FUT1 and GCNT2 function on both glycosphingolipid and glycoprotein substrates, whereas GCNT3 activity is limited to glycoprotein substrates. These results are consistent with a previous study showing that F77 antigen in PC-3 cells is mainly carried by glycolipids (20).

**FIGURE 6.**
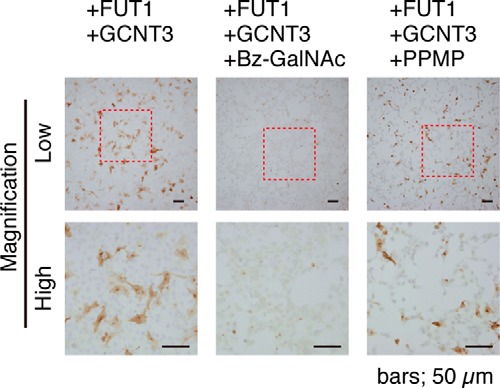
**Effect of glycosylation inhibitors on F77 antigen biosynthesis.** 267B1 cells were transfected with FUT1 plus GCNT3. At 24 h post-transfection, cells were cultured with or without 20 μg/ml PPMP or 5 mm Bz-GalNAc for 48 h. dl-PPMP (Santa Cruz Biotechnology) was used here to inhibit glycosphingolipid synthesis, and Bz-GalNAc was used to inhibit *O-*glycan synthesis. The low magnification images enclosed by the *dotted line* (*upper panels*) were zoomed into high magnification images (*bottom panels*).

In the previous studies, the carrier of F77 antigen in PC-3 cells was determined using two chemical inhibitors, PPMP, an inhibitor of glycosylceramide synthesis, and benzyl-α-GalNAc, an inhibitor of protein *O-*glycosylation. F77 antigen level was decreased in PC-3 cells by PPMP but not by benzyl-α-GalNAc. Here, to determine the carrier of F77 antigen in 267B1 cells, we co-transfected 267B1 cells with FUT1 and GCNT3 and cultured them in medium containing benzyl-α-GalNAc. Such treatment abolished F77 antigen reactivity (Fig. 6). However, when FUT1 and GCNT3 co-transfected 267B1 cells were cultured in medium containing PPMP, F77 antigen levels were unchanged, suggesting that F77 antigen is carried by *O-*glycan glycoproteins in transfected 267B1 cells.

##### F77 Antigen Biosynthesis in CHO and HEK293T Cell Lines

**FIGURE 7.**
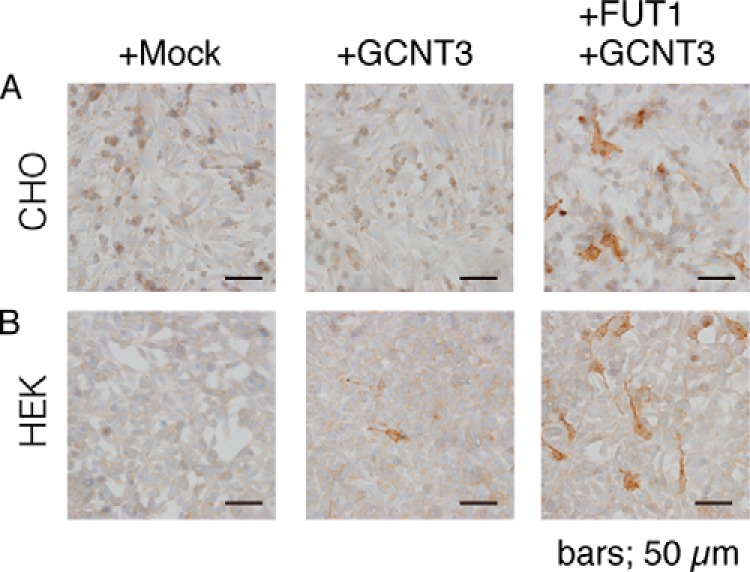
**Immunocytochemistry for F77 in transfected CHO and HEK293T cells.** Both lines were transfected with either GCNT3 only or GCNT3 plus FUT1. At 24 h of post-transfection, cells were fixed and incubated with F77 mAb, followed by incubation with peroxidase-conjugated secondary antibody and the 3,3′-diaminobenzidine color reagent.

As the prostate line 267B1 may express genes encoding factors not included in our GT/ST panel, we next asked if additional gene product(s) were essential for F77 antigen. To do so, we used CHO cells, as they reportedly express a limited set of glycosyltransferases (28). For example, CHO cells do not exhibit detectable α1→3 fucosyltransferase activity (29), and their major cell surface glycans are core-1 *O-*glycan structures (30) and GM3 glycolipids (31). When we co-transfected CHO cells with FUT1- and GCNT3-expressing constructs, cells showed F77 positivity (Fig. 7*A*). We also tested HEK293T cells, a line often used for transfection experiments, and found that GCNT3 alone was sufficient to produce F77 antigen (Fig. 7*B*), most likely because HEK293T cells express FUT1 (see Fig. 5*A*). These results suggest that FUT1 and GCNTs are sufficient for expression of the F77 epitope in a wide range of mammalian cell types.

##### Hypoxia Up-regulates Expression of Glycosyltransferases Required for F77 Epitope Synthesis

**FIGURE 8.**
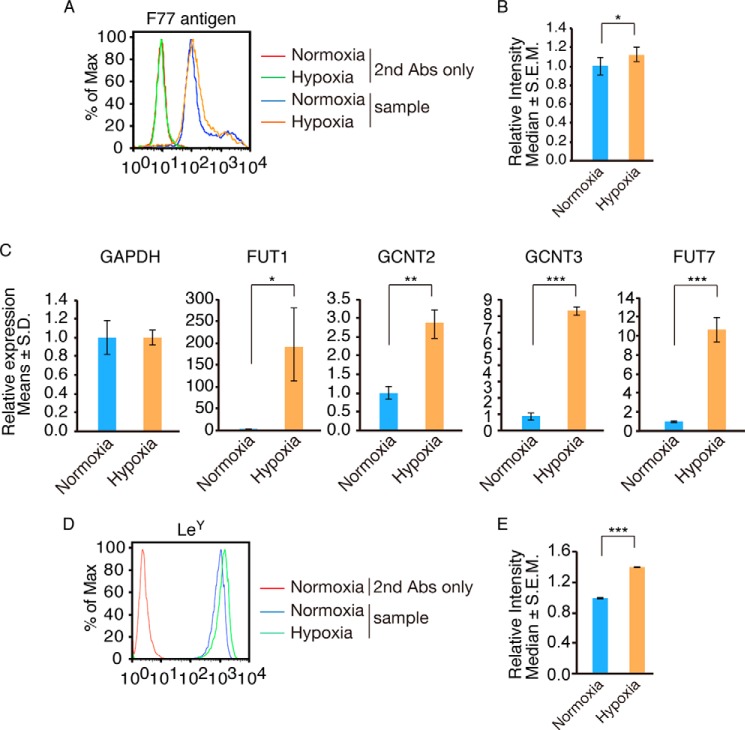
**Effect of hypoxia on expression of FUT1, GCNT2, and GCNT3 in RWPE1 cells.** *A,* flow cytometry of F77 epitope expressed on RWPE1 cells. Cells were cultured under normal conditions (normoxia) or in 95% N_2_, 5% CO_2_ (hypoxia) for 48 h. *Histogram* of RWPE1 cells under hypoxia (*orange line*) was overlaid with normoxia sample cells (*blue line*). Data without F77 are shown in *red* or *green lines. B*, *bar graphs* show quantitative analysis of flow cytometry. *Error bars* indicate S.E. Population comparison was performed using Flowjo software. The experiment was done three times, and they showed similar results. A *p* value less than 0.05 was considered significant (*). *C,* glycosyltransferase transcript levels in RWPE1 cells. Cells were cultured under normoxia or hypoxia for 24 h. GAPDH, FUT1, GCNT2, GCNT3, or FUT7 expression was determined by quantitative RT-PCR using primer sets described under “Experimental Procedures.” *Error bars* indicate S.D. A two-tailed unpaired Student's *t* test was used for statistical analysis. *D*, flow cytometry of Le^Y^ antigen expressed on RWPE1 cells under normoxia and hypoxia. *E*, quantitative analysis of Le^Y^ by flow cytometry. *Error bars* indicate S.E. The experiment was done twice, and they showed similar results. A *p* value less than 0.05 was considered significant (*). **, *p* < 0.01; ***, *p* < 0.001.

Hypoxia reportedly promotes progression of solid tumors and chemoresistance (32) and induces sialyl Lewis A and sialyl Lewis X antigens in colorectal cancer, promoting cancer metastasis (33). To determine whether hypoxia induces F77 epitope expression, we cultured immortalized prostate RWPE1 cells under 95% N_2_, 5% CO_2_ for 48 h. RWPE1 cells expressed F77 antigen moderately under normoxia, but those levels increased slightly under hypoxia (Fig. 8, *A* and *B*). Quantitative RT-PCR demonstrated significant up-regulation of FUT1, GCNT2, and GCNT3 transcript levels in RWPE1 cells cultured under hypoxia for 24 h (Fig. 8*C*). These results suggest that hypoxia induces the three glycosyltransferases involved in F77 antigen synthesis in prostate cells by up-regulating transcription of glycosyltransferases required for its synthesis. The F77 antigen expression is not proportionately increased, possibly due to up-regulation of transcript levels of additional enzymes, resulting in masking of F77 antigen. Indeed, hypoxia increased FUT7 transcript and Le^Y^ antigen levels (Fig. 8, *C–E*), suggesting that α1,3 fucose moiety of Le^Y^ antigen is one of the causes of the masking of F77 antigen.

## DISCUSSION

In this study, we have established that the epitope specifically recognized by mAb F77 is a branched *N*-acetyllactosamine presenting the blood group H antigen as the terminal structure (Fig. 2*B*). This was revealed by co-transfection of a pool of candidate glycosyltransferase expression vectors and subsequent subtractive transfection assays (Fig. 1). We found that FUT1 and at least one glutaminyl (*N*-acetyl)transferase are required for robust F77 antigen positivity. We demonstrated expression of these genes in the F77 antigen-positive prostate cancer lines PC-3 and LNCaP by RT-PCR (Fig. 5*A*). Finally, we confirmed activity of FUT1, GCNT2, and GCNT3 in F77 antigen biosynthesis in loss-of-function experiments using respective siRNAs targeting FUT1 and GCNT2 in PC-3 cells (Fig. 5*B*).

Transfection studies using constructs encoding blood group A and B enzymes suggest that F77 antibody binds less strongly to blood group A and/or B antigens than to the H antigen (Fig. 3). ELISA inhibition assays using synthetic H, A, or B oligosaccharides indicated that any one of the three oligosaccharides blocked F77 antibodies binding to PC-3 cells in a concentration-dependent manner with type H showing the strongest inhibitory effect (Fig. 4). These results suggest that type A and B antigens are bound by F77 but with a lower affinity than type H. These data are in overall accord with the results of TLC blot assays using neoglycolipid-derived bromo-*O-*glycans released from an F77 antigen-positive mucin glycoprotein and microarray analyses with purified glycolipids and neoglycolipids, as described by Gao *et al.* (43). In this study, F77 antigen produced by 267B1 transfectant cells is carried by a glycoprotein via *O-*glycosylation. In contrast, the previous report (20) demonstrated that the majority of F77 antigen expressed by PC-3 cells is expressed on glycolipids. These results indicate that F77 antigen structure is carried on both protein and lipid and is independent of the carrier.

We have also shown that expression of glycosyltransferase genes required for F77 antigen is regulated by conditions associated with malignancy. Among the conditions we examined was hypoxia, a stress commonly detected in rapidly growing solid tumors (34). In hypoxia, multiple genes are induced by hypoxia-inducible transcription factors to overcome oxygen insufficiency. In colon cancer SW488 cells, hypoxia induces expression of *FUT7* and *ST3GAL1*, leading to overexpression of sialyl Lewis X antigen and hematogenous metastasis to liver via E-selectin (33). In malignant colon tumors, high levels of GCNT2 transcripts are found in invasive cells emerging from hypoxic regions (35). In highly metastatic breast cancer lines and tumors, *GCNT2* is overexpressed, an occurrence associated with enhanced cell detachment, adhesion to endothelial cells, migration and invasion *in vitro*, and lung metastasis *in vivo* (36). Elevation of *FUT1*, *GCNT2,* and *GCNT3* expression as seen in RWPE1 cells cultured under hypoxia (Fig. 8) may similarly underlie colon and breast cancer survival responses to hypoxia and promote invasion and metastasis.

Although there has been considerable progress in reducing cancer incidence, prostate cancer is the most prevalent (43%) cancer type among males in the United States (37). Despite the effectiveness of hormone therapy, many patients progress to an androgen-independent and metastatic state, at which time the disease becomes incurable. Therefore, early and accurate diagnosis is crucial for its better prognosis. However, the widely used screening test, which measures prostate-specific antigen levels in blood, often gives false-positive results (38). This is mainly because PSA is secreted in healthy humans, and differing levels of PSA are detected at steady state. A molecular marker that arises only in the cancer condition is desired. At this point, F77 carbohydrate antigen that is specifically synthesized by prostate cancer cells can be a candidate for a novel diagnostic marker. Moreover, it has been shown that targeted mAb therapy has great therapeutic potential as exemplified by the anti-CD20 mAb (rituximab) for B cell lymphoma (39, 40). Several laboratories thus have generated potentially therapeutic mAbs targeting solid tumors, including prostate cancer. mAbs raised against the prostate cancer cell line DU 145 recognize a cell surface antigen expressed on both DU 145 and PC-3 cells and promote cytotoxicity, suggesting their therapeutic potential (41). A cytotoxic ricin-conjugated mAb recognizing a prostate-specific membrane protein inhibits growth of LNCaP tumor xenografts without apparent toxicity to mice (42). This article and the accompanying article (43) have observed that F77 antigen shares the structure of blood group H antigen expressed on normal red blood cells. Further studies are being pursued to determine the utility of F77 in diagnostics or therapies for malignant prostate tumors.

## Footnotes

3 The abbreviations used are:

PPMP: 1-phenyl-2-palmitoylamino-3-morpholino-1-propanol
Bz-GalNAc: benzyl 2-acetamido-2-deoxy-α-d-galactopyranoside
DAB: 3,3′-diaminobenzidine
GT: glycosyltransferases
mAb: monoclonal antibody
ST: sulfotransferase.
